# Variation in the Substitution Rates among the Human Mitochondrial Haplogroup U Sublineages

**DOI:** 10.1093/gbe/evac097

**Published:** 2022-06-22

**Authors:** Sanni Översti, Jukka U Palo

**Affiliations:** Transmission, Infection, Diversification and Evolution Group, Max Planck Institute for the Science of Human History, Kahlaische Straße 10, Germany; Organismal and Evolutionary Biology Research Programme, Faculty of Biological Sciences, University of Helsinki, P.O. Box 56, FI-00014 Helsinki, Finland; Department of Forensic Medicine, Faculty of Medicine, University of Helsinki, P.O. Box 40, FI-00014 Helsinki, Finland; Forensic Chemistry Unit, Forensic Genetics Team, Finnish Institute for Health and Welfare, P.O. Box 30, FI-00271 Helsinki, Finland

**Keywords:** mitochondrial DNA, haplogroup U, ancient DNA, substitution rate variation, tip-calibration, time-dependence, demography

## Abstract

Resolving the absolute timescale of phylogenetic trees stipulates reliable estimates for the rate of DNA sequence evolution. For this end, various calibration methods have been developed and studied intensively. Intraspecific rate variation among distinct genetic lineages, however, has gained less attention. Here, we have assessed lineage-specific molecular rates of human mitochondrial DNA (mtDNA) by performing tip-calibrated Bayesian phylogenetic analyses. Tip-calibration, as opposed to traditional nodal time stamps from dated fossil evidence or geological events, is based on sample ages and becoming ever more feasible as ancient DNA data from radiocarbon-dated samples accumulate. We focus on subhaplogroups U2, U4, U5a, and U5b, the data including ancient mtDNA genomes from ^14^C-dated samples (*n* = 234), contemporary genomes (*n* = 301), and two outgroup sequences from haplogroup R. The obtained molecular rates depended on the data sets (with or without contemporary sequences), suggesting time-dependency. More notable was the rate variation between haplogroups: U4 and U5a stand out having a substantially higher rate than U5b. This is also reflected in the divergence times obtained (U5a: 17,700 years and U5b: 29,700 years), a disparity not reported previously. After ruling out various alternative causes (e.g., selection, sampling, and sequence quality), we propose that the substitution rates have been influenced by demographic histories, widely different among populations where U4/U5a or U5b are frequent. As with the Y-chromosomal subhaplogroup R1b, the mitochondrial U4 and U5a have been associated with remarkable range extensions of the Yamnaya culture in the Bronze Age.

**Significance**

Mitochondrial DNA (mtDNA) variation carries resolvable signals of events in the history of a population, but the absolute timescale of these events can only be attained if the rate of sequence evolution can be estimated. Molecular rate estimates obtained with different calibration methods are known to vary, but little is known about rate differences between distinct human mtDNA lineages. Here, we have estimated molecular rates specific to four mtDNA lineages belonging to haplogroup U using both ancient and modern mtDNA data and clock calibration based on sample ages (“tip-calibration”). The results suggest, for the first time, variation in the molecular rates between the U lineages, which could derive from differences in population history.

## Introduction

The last decade has seen a remarkable increase in the availability of modern and ancient genetic data from humans and other organisms. This, together with the parallel increase in computing power, has made molecular inferences of our past easier and more attractive for researchers. Yet, the accuracy of most of these molecular inferences hinges on the rate of molecular evolution assumed in the analyses. As noted in Endicott and Ho 2008 “understanding the time-frame of human evolution and migration is one of the most prominent goals of genetic analysis.” (Endicott and Ho 2008).

Molecular dating has become extensively exploited since the introduction of molecular clocks in the 1960s (e.g., Margoliash 1963). By assuming a certain mutation rate per time unit, the number of mutations observed between sequences can be translated into calendar time. Retrieval of this time-bounded molecular rate is not necessarily straightforward, though, as it requires calibration of the molecular clock with external temporal information. Traditionally molecular rates have been calibrated using dated fossil evidence, providing the lowest age bound for the appearance of the species (see e.g., Donoghue and Benton 2007). Similarly, a dated geological or environmental event could act as a calibration point (for review, see Ho et al. 2015). One widely used geological calibration point is the formation of the Isthmus of Panama c. 3 MYA that prevented transoceanic migration of aquatic organisms but connected terrestrial species and populations of the two American subcontinents (O’Dea et al. 2016). Additionally, the number of molecular differences in successive generations could be recorded in pedigree studies and this so-called pedigree rate can then be implemented in phylogenetic analyses. (For review of different calibration methods, see Ho et al. 2011; Box 1.)

Along with a rapidly growing field of ancient DNA (aDNA) research, more calibration mechanisms have become available. The archaeological samples used as a source of aDNA can be dated, for example, using the decay of radiocarbon (^14^C) and these sample ages can be entered in phylogenetic analyses as “tip-dates” to resolve the molecular rate. Tip-dates can be heterochronous, including, for example, different sampling points of viruses or ages of archaeological samples. Certain phylogenetic methods, such as those incorporated in the widely used the BEAST 2 software package (Bouckaert et al. 2019), also allow incorporation of the uncertainty in the tip-date, such as the probability distributions of radiocarbon dates. However, it has been shown that for a sample set including ^14^C-dated samples covering a comprehensive timespan, accounting for the uncertainty in the dating have only minor impact in the divergence date estimates (Molak et al. 2013, 2015; for a general review see Ho et al. 2011 and Bromham et al. 2018).

Since node calibration is associated with considerable ambiguity and, in case of human mitochondrial DNA (mtDNA), usually impossible without a more distant outgroup, exploiting tip-calibration has shown to result in more accurate and consistent outcomes (Rieux et al. 2014). Substantial time-dependency is emerging as evident among the molecular rates: in humans, for instance, pedigree-based rates for mtDNA are considerably higher than the substitution rates deduced from the long-term phylogenetic analyses (Stoneking et al. 1992; Forster et al. 1996; Parsons et al. 1997; Henn et al. 2009). Additionally, human mtDNA rates evaluated from the ancient samples are in-between the long-term and pedigree-based estimates (Fu et al. 2013; Rieux et al. 2014, see also Ho et al. 2007). This implies that the magnitude of the rate is heavily dependent on the length of the time interval in scrutiny (Ho et al. 2005). (For a review of observed time dependent molecular rates, see Box 2 in Ho et al. 2011.) The differences in pedigree-based and phylogenetic mutation rate estimates stem from the actions of selection and drift: the longer the time-frame the smaller the proportion of spontaneous mutations surviving in the gene pool (Ho et al. 2011).

Whereas the discrepancies among the molecular rates recovered with the different calibration methods have been the target of intensive research, less attention has been paid on the rate variation among the distinct lineages. In humans, haplogroup-specific deviations have been characterized in the Y-chromosomal evolutionary rates (Dupuy et al. 2004; Claerhout et al. 2018): the elevated rate of haplogroup R1b have been correlated with the relatively recent rapid spread of the lineage in Europe (Larmuseau et al. 2014; Solé-Morata et al. 2014). Instead, for the human mitochondrial genomes, the distinct mutation rates for some individual lineages in present-day populations have been interpreted to be a consequence of differences in the selective pressures among haplogroups (Torroni et al. 2001; Pierron et al. 2011).

In addition to selection, the time-dependency of substitution rates is also influenced by drift (Ohta 1987, 2002, see also Bromham and Penny 2003). This is particularly interesting as it links the substitution rate to the demographic history of a population. As the frequencies of mtDNA lineages vary between human populations this also hints to the possibility of lineage-specific variation in substitution rates. Studies focusing on tip-calibrated lineage-specific variation are, to our knowledge, thus far scarce (but see Brotherton et al. 2013). This is rather surprising, as interlineage substitution rate variation can affect divergence time estimates, demographic events and effective population size estimates, that is, many central results in population genetic assessments.

Here, we have tested the hypothesis of lineage-specific mutation rates by performing tip-calibrated phylogenetic analyses in a Bayesian framework for the sublineages of human mitochondrial haplogroup U. Haplogroup U was chosen here, since its main subhaplogroups have undergone comparatively distinct population dynamics in the prehistory (see Discussion), making it feasible to assess the impact of demographic past into the substitution rate. Haplogroup U, common among contemporary west Eurasians, is one of the earliest haplogroup found from the archaeological human remains in Europe, with the oldest observations dating back ∼38,000 years (Krause et al. 2010). Hence, the prevalence of U (in the European mitochondrial genepool) covers an extensive timespan and the availability of ^14^C-dated archaeological samples carrying haplogroup U is abundant.

## Results

### MtDNA Haplogroup U Genomes

**Fig. 1.**
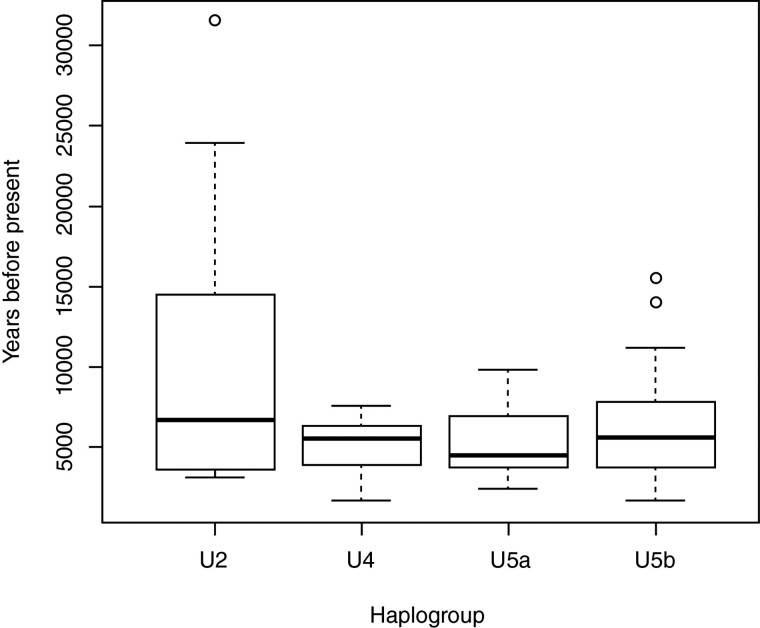
Distribution of the mean values of ^14^C dates among the ancient samples per subhaplogroups. Bolded bars within boxes display median values and boxes represent upper and lower quartiles. Whiskers illustrate the lowest and highest values whereas circles show the outliers.

Altogether 535 complete mitochondrial genomes from databases and from previously published articles were included in the analyses. This material included three different data sets: 1) Ancient mtDNA genomes (*n* = 234), 2) Contemporary mtDNA sequences (*n* = 301), and 3) Outgroup (“R-outgroup”). Most of the ancient hg-U mtDNA sequences were collected from the Ancient mtDNA database (AmtDB, Ehler et al. 2019) and additional samples were obtained from publications (see supplementary table S1, Supplementary Material online). Since the aim of this study was to evaluate the substitution rates using tip-dating, only sequences produced from ^14^C-dated samples were included. Furthermore, all sequences with >10% of missing nucleotides were excluded. Table 1 shows the number of sequences included per haplogroup, more detailed information of the samples (such as subhaplogroup, ^14^C date and percentage of missing data) can be found in supplementary table S1, Supplementary Material online. The distributions of ^14^C dates per haplogroup are represented in figure 1. For the contemporary samples haplogroups and GenBank IDs are presented in supplementary table S2, Supplementary Material online.

**Table 1:** Number of Samples Per Subhaplogroup

	Ancient (*n*)	Contemporary (*n*)
U2	19	42
U4	42	62
U5a	99	99
U5b	74	98

Contemporary sequences were obtained from PhyloTree v17 (van Oven and Kayser 2009). For each of the subhaplogroup (of U2, U4, U5a, and U5b), one contemporary sequence was chosen. This approach is dictated by limitations of the Bayesian approach that we use in this study. This artificial selection of one haplotype (from each subhaplogroup) obviously does not represent the real frequencies of haplotypes and hence the resulting set of sequences is not a random sample from the sequence pool. This non-representative sampling should not bias the substitution rate estimates the same way it has been shown to bias effective population size estimates (Kuhner 2009).

To evaluate the impact of presence of an outgroup in the analyses, two Paleolithic samples representing haplogroup R were used. These samples included Fumane 2 dating back to ∼39,805 calibrated years before present (calYBP) (GenBank ID: KP718913 [Benazzi et al. 2015]) and Ust′-Ishim dating back ∼45,050 calYBP (Fu et al. 2014). These two samples are later in the text referred to as “R-outgroup.”

**Fig. 2.**
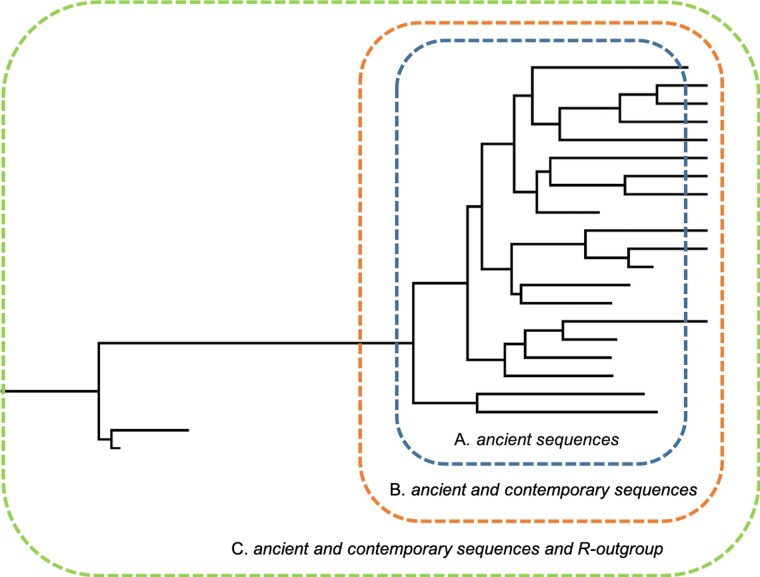
Schematic illustration of different data sets used in three distinct scenarios. (*A*) Blue = analysis containing only aDNA sequences, (*B*) orange = analysis containing aDNA sequences and contemporary sequences, and (*C*) green = analysis containing aDNA sequences, contemporary sequences, and R-outgroup.

For subhaplogroups of U, three distinct analyses were performed, containing A) Only aDNA sequences, B) aDNA sequences and contemporary sequences, and C) aDNA sequences, contemporary sequences, and R-outgroup (see fig. 2). As analyses were executed separately for each subhaplogroup, in total we carried out 12 independent analyses.

### Substitution Models Chosen

To select the most suitable substitution model, we used a model averaging tool bModelTest (Bouckaert and Drummond 2017) implemented in BEAST 2. For all the other datasets, highest posterior support was obtained for Hasegawa, Kishino & Yano (HKY) model (Hasegawa et al. 1985) except for U5b, for which the general time reversible (GTR) (Tavaré 1986) model was supported (supplementary table S3, Supplementary Material online). For each dataset, the gamma-distributed rate heterogeneity (+Γ) and proportion of invariant sites (pInv) were taken into account.

For two analyses of U2 (aDNA + contemporary and aDNA + contemporary + R-outgroup), the original substitution model proposed by bModelTest (GTR + Γ + pInv) turned out to cause poor mixing of MCMC chains, presumably due to the huge number of parameters involved in the model. To achieve a sufficient sample of all possible parameter value combinations, HKY + Γ + pInv mutation model was used. To avoid the possible bias introduced by the simpler model, the similarity of the posterior distributions for the parameters of interest (i.e., *ucldMean, ucldStdev, TreeHeight, pInv, gammaShape*) between these two models were evaluated by eye. Since the distributions were highly overlapping or identical, HKY + Γ + pInv was used in the subsequent analysis for scenarios B and C of U2.

### Molecular Rate Estimates

Since tip-dates of heterochronously sampled sequences were used as a only source for calibration, we used TempEst (Rambaut et al. 2016) to evaluate if the sampling dates were sufficient enough to produce a temporal signal. All data sets showed a positive correlation between genetic divergence and sampling time (see supplementary table S4, Supplementary Material online). For all the haplogroups for each scenario, *R*^2^ values ranged from 0.10 to 0.84. In Rambaut et al. (2016) similar values were considered adequate for downstream analyses. According to the results, the temporal signal embedded in the data is sufficient for further phylogenetic molecular clock analysis.

**Fig. 3.**
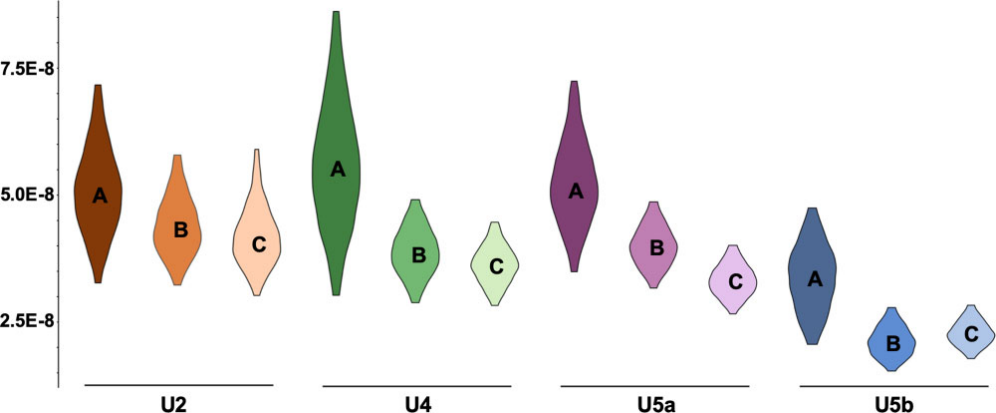
Molecular rates for the different haplogroups under scenarios A, B, and C. Scenario A: only aDNA sequences, scenario B: aDNA sequences and contemporary sequences, and scenario C: aDNA sequences, contemporary sequences, and R-outgroup. Distributions represent 95% highest posterior density of the molecular rates (*ucld.Mean*). All values are ×10^−8^ substitutions/site/year. For detailed information see supplementary table S5, Supplementary Material online.

Molecular rates were estimated for sublineages of U, by using three different datasets: the first scenario included only ancient sequences (A), second contained ancient and contemporary sequences (B) whereas the third comprised in addition to the ancient and contemporary samples also an outgroup (R-outgroup; scenario C, see fig. 2). Results for the molecular rates of each sublineage (U2, U4, U5a, and U5b) are presented in figure 3 (see also supplementary table S4, Supplementary Material online).

For all U sublineages, the mean substitution rate determined based solely on aDNA sequences (scenario A), was higher than the two other estimates (scenarios B and C; fig. 3, supplementary table S5, Supplementary Material online). Within subhaplogroups the mean molecular rate obtained from ancient sequences only (scenario A) was approximately 1.2–1.6 times higher than the rates determined based on two other scenarios (B and C). The rates obtained for scenarios B and C were rather similar to each other in all subhaplogroups (B:C = 0.9 … 1.2). Largest differences were observed for U4 and U5a: the rate estimate for scenario A was 1.3–1.4 times higher than the estimate for scenario B and further it was 1.6 times higher than for scenario C. For lineage U5b, the rate assessed with the outgroup (scenario C) yielded a marginally higher point estimate than without the outgroup (C:B = 1.07), whereas for U2, U4, and U5a the rooted estimate was somewhat lower (C:B = 0.82 … 0.97). However, inclusion of R-outgroup did not have a notable influence on the molecular rate estimate.

In all three scenarios, differences in the substitution rates were observed between subhaplogroups. Since rate estimates for scenarios B and C (aDNA + contemporary and aDNA + contemporary + R-outgroup) were highly overlapping for each lineage, only scenario B is discussed below. Whereas in scenarios A and B, the mean estimates for U2, U4, and U5a were largely in accordance, subhaplogroup U5b stands out by having notably lower mean estimates than the other subhaplogroups. The largest differences are seen in scenario B, where the rates for U2 and U5a are 2.1 and 1.9 times higher than for U5b, respectively (fig. 3 and supplementary table S5, Supplementary Material online). However, it has to be noted that the 95% highest posterior density (HPD) intervals are largely overlapping suggesting non-significant differences between values.

### Dependability of the Rate Estimates

In general, tip-calibration has been previously used to determine substitution rates for human mtDNA (Brotherton et al. 2013; Fu et al. 2013; Rieux et al. 2014). Estimates in Fu et al. (2013) and Rieux et al. (2014) were determined for datasets containing various haplogroups, whereas in Brotherton et al. (2013) the rate was obtained for ancient sequences belonging to mitochondrial haplogroup H. The rates obtained in these studies were rather similar ranging between 2.14 and 2.67 (× 10^−8^ substitutions/site/year), and close to our estimates for subhaplogroup U5b.

A substitution model can have a significant impact on molecular rate estimates. As for all the other subhaplogroups the best-fit substitution model was HKY + Γ + pInv, but a more complex model (GTR + Γ + pInv) turned out to fit better for U5b. To evaluate if the lower molecular rate observed in U5b could result purely from the usage of different substitution models, we performed parallel analysis for U5b with HKY + Γ + pInv. The rate estimates for both HKY + Γ + pInv and GTR + Γ + pInv are, however, nearly identical (supplementary table S5, Supplementary Material online). In addition, since it has been shown that at intraspecies level modeling rate heterogeneity among sites with proportion of invariant sites might bias the evolutionary rates and divergence time estimates (Jia et al. 2014), we performed further BEAST analysis for scenarios A and B without pInv. As shown in supplementary table S6, Supplementary Material online, molecular rate estimates are robust for inclusion/exclusion of pInv and thus the rate variation between lineages cannot be attributed to the use of different substitution models.

When dealing with aDNA data, the sequence quality is always a potential source of error. To minimize the impact introduced by poor sequence quality, we included only aDNA sequences with less than 5% of missing data. In addition, the distributions of ancient sample ages in each of the subhaplogroups were similar (see fig. 1) and all subhaplogroups had ancient sequences with roughly similar proportions (40–50%, U2 lowest with 31%). Even though post-mortem damage appears to have only limited effect on molecular rates (Rieux et al. 2014), the positive correlation between genetic divergence and sampling times observed with TempEst further confirms that ancient sequences used in this study do not contain considerable levels of dating errors and/or post-mortem modifications.

To further exclude sequence degradation as a significant causal agent for the molecular rate differences, we compared the coefficient of variation, a parameter describing the clock-likeness of the data, estimates for contemporary-only data with estimates obtained for scenarios A and B (aDNA-only and aDNA + contemporary, respectively). For this end, we conducted additional BEAST 2 analyses for contemporary-only data by using an uncorrelated lognormal relaxed clock model and Bayesian skyline plot as a tree prior. To calibrate the phylogenetic trees containing only contemporary sequences, we used subhaplogroup age estimates obtained in Behar et al. 2012 as a tMRCA prior [in years for U2 N(42,805; 4,493), U4 N(17,493; 3,069), U5a N(22,440; 4,926), and U5b N(22,794; 3,590)]. We then evaluated the marginal posterior distributions of coefficient of variation obtained for the different data sets. No stringent value threshold for model choice can be given, but values below 0.1 are most often interpreted as a support for the usage of a strict clock model. Results are presented in supplementary table S8 and figure S1, Supplementary Material online. For lineages U2, U4, and U5b coefficient of variation values and posterior distributions are nearly identical within each haplogroup for all different datasets (contemporary-only, aDNA-only, aDNA + contemporary). This indicates that lineages U2, U4, and U5b have evolved, more or less, in a clock-like fashion. Together with the TempEst results, the congruence between ancient-only and contemporary-only estimates for lineage U5b, implies that rate variation among haplogroups cannot be explained by differences in the aDNA damage patterns between different haplogroups.

Instead, the higher coefficient of variation value for U5a aDNA-only data suggests that U5a has evolved in a less clock-like manner compared to the other haplogroups. In general, it is extremely difficult to imagine that bias caused by sequencing errors and/or DNA degradation are affecting only one subhaplogroup (U5a), especially since U5a sequences originate from different studies including sequences belonging to other subhaplogroups and included in this study.

Despite the strong evidence for the validity of lower substitution rate in U5b, we performed additional strict clock analysis with BEAST 2 for scenarios A and B (aDNA and aDNA + contemporary, respectively). As shown in the supplementary table S7, Supplementary Material online, even when implementing the strict clock the previously observed pattern remains: haplogroup U5b has notably lower molecular rate than three other U lineages for both scenarios.

To further confirm our results with an independent approach, we performed additional analyses with Least-Squares Dating (LSD2) method v.1.9.9 (To et al. 2016) implemented in IQ-Tree 2.0.3 (Minh et al. 2020) for scenarios A and B. Similarly, to BEAST 2 analyses, with LSD scenario A yields higher estimates than scenario B in each subhaplogroup (supplementary table S9, Supplementary Material online). In addition, we observe differences between haplogroups: U2 showed the highest values whereas U5b exhibits the lowest point estimates. However, differences between haplogroups are not as outstanding as the discrepancy seen with the Bayesian approach. Plausible explanations for slight differences between these two methods are that LSD assumes a strict clock and does not directly take into account phylogenetic uncertainty.

Furthermore, since a previous study has found evidence of negative selection for human mitochondrial subhaplogroups U5b1, U5a1d, and U4d (Malyarchuk 2011), it was necessary to assess the possible differences of selective influence between the lineages in our data. The Z-tests implemented in MEGA-X revealed similar signals of negative selection in all haplogroups, regardless of the data set used (scenarios A, B, and C; see supplementary table S10, Supplementary Material online). The observed similarity of the selection signals between haplogroups does not lend support for the marked role of selection in shaping the lineage-specific substitution rates.

### Comparison of Divergence Time Estimates with Previously Published Estimates

We then evaluated the effect of sublineage-characteristic substitution rates on the estimates of divergence times. Special attention was paid to the U5, given the notable discrepancy in the substitution rates between U5a and U5b. For all U subhaplogroups (U2, U4, U5a, and U5b), comparison was performed with estimates presented in Soares et al. (2009) and Behar et al. (2012). Additionally, for U5a and U5b values were compared with Malyarchuk et al. (2010) since that is the most comprehensive study focusing on the dating of U5 and its sublineages. For the comparison, we used the divergence time estimates determined based on scenario B (U + contemporary). Results are presented in table 2.

**Table 2:** Comparison of divergence time estimates for Hg-U subhaplogroups between this study and Soares et al. (2009), Malyarchuk et al. (2010) and Behar et al. (2012). All the values are thousand years ago (kya). For this study, median values are presented with 95% highest posterior density intervals. For Soares et al. (2009) and Malyarchuk et al. (2010) 95% confidence intervals are presented. For Behar et al. (2012) lower and upper bounds are calculated based on standard deviation provided in the original publication.

Haplogroup	This study	Soares et al. 2009	Behar et al. 2012	Malyarchuk et al. 2010
U2	39.8 [38.0, 44.6]	53.5 [40.3, 67.2]	42.8 [38.3, 47.5]	–
U2a	17.6 [9.8, 26.3]	27.5 [13.2, 42.8]	22.7 [14.4, 31.0]	–
U2b	12.0 [7.0, 18.5]	34.3 [22.3, 46.9]	29.3 [23.5, 35.1]	–
U2c	14.1 [8.8, 19.8]	34.8 [22.3, 47.9]	29.9 [23.3, 36.5]	–
U2e	17.2 [13.1, 21.5]	16.7 [9.9, 23.8]	19.3 [15.2, 23.4]	–
U2d	13.1 [8.6, 18.7]	–	20.8 [15.7, 25.9]	–
				
U4	16.9 [12.9, 23.1]	20.9 [11.0, 31.2]	17.5 [14.4, 20.6]	–
U4a	13.1 [10.5, 16.3]	–	14.9 [11.6, 18.2]	–
U4a1	10.9 [8.9, 13.1]	–	7.7 [5.1, 10.3]	–
U4a2	12.0 [9.8, 15.0]	–	8.8 [6.3, 11.3]	–
U4b	13.4 [10.8, 16.3]	–	12.6 [9.8, 15.4]	–
U4b1	12.5 [10.4, 15.5]	–	11.5 [8.7, 14.3]	–
U4d	11.6 [9.3, 14.7]	–	14.9 [11.3, 18.5]	–
				
U5a	17.7 [14.1, 22.5]	26.9 [16.1, 38.1]	22.4 [17.5, 27.3]	19.9 [13.3, 26.1]
U5a1	15.0 [12.5, 17.7]	18.2 [9.8, 27.1]	16.9 [14.1, 19.7]	16.2 [11.8, 20.7]
U5a1a	9.1 [6.8, 11.5]	–	12.1 [8.1, 16.1]	–
U5a1a1	7.3 [5.7, 9.4]	–	6.8 [2.9, 10.7]	12.3 [5.4, 19.5]
U5a1a2	7.9 [5.8, 10.2]	–	10.3 [6.7, 13.9]	–
U5a1b	7.6 [5.7, 10.0]	–	8.4 [5.6, 11.2]	11.2 [6.8, 15.7]
U5a1c	13.6 [11.5, 15.3]	–	14.6 [10.6, 18.6]	13.0 [6.3, 19.9]
U5a1d	11.8 [9.5, 14.6]	–	15.1 [11.9, 18.3]	19.0 [10.5, 27.9]
U5a2	15.4 [12.9, 17.7]	22.0 [11.5, 33.1]	18.4 [14.5, 22.3]	14.4 [9.1, 19.9]
U5a2a	13.9 [12.3, 15.6]	–	13.0 [7.1, 18.9]	5.7 [3.4, 8.0]
U5a2b	10.6 [7.7, 13.6]	–	11.4 [8.2, 14.6]	8.3 [6.0, 10.6]
U5a2c	13.8 [12.1, 15.4]	–	11.4 [8.1, 14.7]	12.8 [6.6, 19.3]
U5a2d	13.1 [10.9, 14.7]	–	16.9 [13.1, 20.7]	–
				
U5b	29.7 [22.8, 31.7]	27.4 [19.4, 35.6]	22.8 [19.2, 26.4]	23.8 [17.7, 31.1]
U5b1	21.8 [16.7, 30.7]	24.0 [16.4, 31.9]	15.5 [10.6, 20.4]	17.7 [9.8, 23.9]
U5b1b	17.5 [12.4, 22.0]	–	10.8 [6.5, 15.1]	–
U5b1c	15.3 [10.4, 19.9]	–	10.4 [7.1, 13.7]	12.8 [5.9, 20.0]
U5b1d	15.7 [11.3, 19.9]	–	11.7 [7.4, 16.0]	–
U5b2	25.5 [21.2, 32.1]	22.4 [14.9, 30.2]	20.0 [16.2, 23.8]	23.7 [16.6, 31.2]
U5b2a	20.9 [15.9, 26.6]	–	14.9 [11.2, 18.6]	19.9 [13.1, 27.0]
U5b2b	22.2 [18.7, 26.7]	–	14.7 [12.0, 17.4]	19.0 [12.1, 26.1]
U5b2c	17.2 [12.3, 21.6]	–	12.7 [7.0, 18.4]	–
U5b3	16.2 [11.2, 23.4]	4.3 [1.2, 7.5]	10.5 [7.6, 13.4]	10.6 [5.0, 16.4]

For certain subhaplogroups of U2, the divergence time estimates reported in this study are based on comparatively small sample sizes (for U2a N = 3, U2b N = 3, U2c N = 4, and U2d N = 5) and hence the divergence time estimates might not be comparable with the dates presented in Soares et al. (2009) and Behar et al. (2012). Nevertheless, the age estimate for U2 [39,800 ybp 95% HPD (38,000; 44,600)] overlaps with the previous estimates. For U4 and its subhaplogroups, dates presented in this study are in agreement with Behar et al. (2012) estimates.

Whereas in the previous studies the divergence time estimates for subhaplogroups U5a and U5b are comparably analogous within studies (table 2) distinguishable differences are visible in this study, U5b being even more than 10,000 years older than U5a. Thus, the divergence estimate presented in this study for U5a is lower compared to the earlier studies, whereas age for U5b appears to be somewhat older than estimates in Soares et al. (2009), Behar et al. (2012) and Malyarchuk et al. (2010).

## Discussion

Dating of the human mitochondrial tree relied at the early stages on the human-chimpanzee split, various biogeographical and archaeological calibrations as well as substitution rates observed in pedigree studies (see Endicott and Ho 2008 and references therein). These different calibration methods have resulted in large discrepancies in the ages estimated for the most recent common ancestor (MRCA) of human mitochondrial genomes (see fig. 1 in Ho and Endicott 2008). Furthermore, a clear positive correlation exists between the age of the calibration point and the estimated age of the MRCA (Ho and Endicott 2008).

Instead of the traditional internal node calibration, the advent of DNA data from ancient, radiocarbon-dated samples has allowed tip-calibration. Obviously, the feasibility of tip-calibration depends on the availability of samples old enough to allow subsequent accumulation of mutations. Studies comparing tip-dating and internal node calibration (Gilbert et al. 2008; Rieux et al. 2014) have suggested that tip-calibration gives more consistent results (Rieux et al. 2014).

Here we present a comparison of relative mtDNA substitution rates, obtained by tip-dating for the human haplogroup U sublineages. Instead of focusing on absolute rates, we aim to prove interlineage mutation rate differences that could affect the timescales of evolutionary events commonly inferred in phylogenetic analyses.

### Time-Dependency Shown With Different Data Sets

Within all the Hg-U sublineages, the rates estimated exclusively based on the ancient sequences (scenario A), were elevated compared to the estimates obtained from analyses including both ancient and contemporary sequences (with or without an outgroup, scenarios B and C, respectively). This complies with simulated results reported in Ho et al. (2007): data sets containing only aDNA sequences gave substitution rates that were higher than those obtained by long-term phylogenetic analyses. Moreover, the rates estimated based on ancient sequences show higher uncertainty than the estimates from other data sets (cf. fig. 3), which also complies with the simulated results in Ho et al. (2007). This likely arises from the lower information content in data sets comprising only ancient sequences.

The observed difference has been interpreted to result from time-dependency, that is, that the estimated rates rely heavily on the length of time interval in question, longer time periods producing lower substitution rates (Ho et al. 2005). In this study the estimates obtained from scenario C, covering the longest time interval, presumably reflect the long-term phylogenetic rates describing the changes in the number of fixed mutations.

Correspondingly, the differences between the rates obtained in this study and previous tip-calibrated estimates can be explained with the differences in time interval focused on. In Rieux et al. (2014) the substitution rate was determined based on the ancient and contemporary sequences with wide geographical distribution, representing virtually all the main maternal haplogroups among present-day populations. Similarly, in Fu et al. (2013), the analyzed ancient samples originating from Europe and Eastern Asia included sequences from both macrohaplogroups M and N. The wide coverage of human mtDNA variation in these studies obviously means longer evolutionary timescale under scrutiny.

### Lineage-Specificity in Mutation Rates

In addition to the differences in substitution rates between data sets, we observed substantial interlineage rate differences. Here, especially subhaplogroup U5b stands out in yielding a considerably lower molecular rate than either U2, U4, or U5a. This difference is also reflected in the subhaplogroup divergence time estimates: whereas earlier studies (Soares et al. 2009; Malyarchuk et al. 2010; Behar et al. 2012) have obtained different ages for U5 subhaplogroups, within each study the ages for U5a and U5b have been relatively similar. However, the ages estimated here for U5a and U5b were widely different: 17,700 and 29,700 ybp, respectively.

It is very difficult to imagine that this difference would derive from disparities in spontaneous mutation rates between lineages. Whereas some sequences, like polynucleotide stretches and GC-rich sequences, are more mutation-prone than others (e.g., Aggarwala and Voight 2016), the relatively minor sequence differences between, for example, U5a and U5b genomes are very unlikely to instigate mutation, for instance, through conformational changes. Therefore, it is far more probable that the observed differences come from dissimilar fractions of spontaneous changes that have become fixed in the gene pool and as such due to population-level factors like selection and drift. Both of these forces can simultaneously modify variation within populations in a similar manner, but their relative importances may, however, be difficult, if not impossible, to isolate.

In the case of mtDNA, the effect of natural selection on human mtDNA variation is still unclear, despite a great number of studies. The results have been contradictory, some showing evidence for directional selection of certain mtDNA encoded proteins, some advocating neutrality (see Kivisild et al. 2006 and references therein). Several studies speaking for the role of selection have also found evidence for lineage-specific (e.g., Malyarchuk 2011) and region-specific differences in mtDNA selection (Mishmar et al. 2003; Ruiz-Pesini et al. 2004). The regional differences have been proposed to stem from adaptive pressures posed by climate, which appears plausible given the role of mtDNA in the energy production of cells. However, these studies have failed to reveal a consistent pattern, and for most findings conflicting results have also been presented excluding the possibility of climate being the only selective influence (Moilanen et al. 2003; Elson et al. 2004; Kivisild et al. 2006, but see Balloux et al. 2009). As a whole this suggests that, while the role of selection cannot be excluded completely, it is unlikely to be the main force in shaping the mitochondrial diversity.

In the current data sets, we observed similar signals of (negative) selection in all subhaplogroups. Although the similar signal of negative selection observed in all subhaplogroups is, as such, an interesting result warranting further studies, it cannot explain the observed molecular rate differences between haplogroups. In fact, it is rather difficult to envisage how the slight differences in the mtDNA genomes of different subhaplogroups would convey significant selection advantages over the others.

### Other Potential Causes for Rate Variation among Lineages

Apart from selection discussed above, the observed rate variation among the human mtDNA lineages can, in theory, also derive from several biasing factors in the samples, data or in the phylogenetic analyses. We can think of five such factors: 1) sequence quality, that is, differing DNA damage patterns among lineages, 2) uneven phylo-temporal distribution of ancient samples analyzed (Rieux and Balloux 2016; Tong et al. 2018), 3) different substitution models chosen (U5b: GTR, others: HKY), 4) proportion of invariant sites assumed in the analyses, and 5) clock model assumed (strict vs. relaxed).

First, factors related to sampling and sequence quality (1–2 above) may bias the results. In old archaeological samples post-mortem nucleotide alterations and strand fragmentation could lead to mutation artifacts (Pääbo 1989; Sawyer et al. 2012), which could explain the results if haplogroup-specific differences in sample (sequence) quality existed. Particularly the deamination of cytosine residues leads to an excess of nucleotides A and T, which in turn might lead in overestimation of the mutation rate, although these post-mortem modifications have been shown to have only limited influence on the substitution rates (Rieux et al. 2014). Ancient samples often yield partial data due to low quality and/or quantity of endogenic DNA. To avoid the possible bias arising from the incomplete sequences, only samples with less than 5% of missing data were included in the study. The impact of such a level of missing data in BEAST analyses have been shown to be small when estimating the times of divergence events (Zheng and Wiens 2015).

We think that we can safely exclude sequence degradation as a significant causal agent for the lower mutation rate in U5b. As post-mortem degradation affects only aDNA sequences, it should lead to very divergent rate estimates for the ancient and contemporary sequences. This was not observed: coefficient of variation estimates were nearly identical for contemporary-only and ancient-only data sets in U2, U4, and U5b. Furthermore, post-mortem modifications would bias the relationship between sample age and genetic divergence, which we did not observe: in each subhaplogroup the TempEst analyses revealed positive correlation between the sample age and genetic differences.

In the above-mentioned analyses, a higher coefficient of variation value was obtained for U5a aDNA-only data, suggesting less clock-like evolution in U5a than in the other haplogroups. While this cannot explain the rate difference in U5b, it is also unlikely to derive from haplogroup-specific sequencing errors and/or DNA degradation. U5a sequences included in the present study originate from different studies that also produced sequences that are included in the other subhaplogroups analyzed here.

Second, the data analysis (model) parameters (3–5 above) could bias the rate estimates. We couldn’t find any evidence for this, either. The analyses were repeated using alternative parameter assumptions: using the same substitution model (HKY) for all lineages, with and without taking the proportion of invariants into account and using both strict and relaxed clocks. None of these analyses produced any qualitative change in the results: haplogroup U5b showed significantly lower substitution rate regardless of the model parameters.

All this leaves drift as the only likely agent behind the substitution rate differences, thus linking them closely to the demographic histories of populations carrying these subhaplogroups. Indeed, based on the aDNA and the distributions among contemporary populations, it is evident that U5a and U5b have undergone comparatively different demographic pasts, especially since the beginning of the Bronze Age.

### Different Demographic Trajectories of Hg-U Sublineages

Haplogroup U is one of the oldest human mitochondrial lineages in Europe, which probably arose around 45,000–55,000 years ago (ya) (Soares et al. 2009; Behar et al. 2012). Earliest U lineages retrieved from late Pleistocene hunter-gatherers represent subhaplogroups U2 (38,000 ya), U8 (33,000 ya), and U5 (31,100 ya) (Krause et al. 2010; Fu et al. 2013; Posth et al. 2016). Around 14,500 ya, after the Last Glacial Maximum (LGM), U5a and U5b became the most prevalent mitochondrial lineages in Europe replacing U2 and U8 partially (Posth et al. 2016). Presumably, U5a and U5b evolved already during the LGM and subsequently expanded from glacial refugia following the continental ice-sheet retreat (Malyarchuk et al. 2010).

During the Mesolithic Stone Age, U5a was characteristic especially for the Baltic and East European hunter-gatherers (Bramanti et al. 2009; Krause et al. 2010; Der Sarkissian et al. 2013; Haak et al. 2015; González-Fortes et al. 2017; Mathieson et al. 2018; Mittnik et al. 2018) and was relatively common also in Scandinavia (Malmström et al. 2009, 2015; Skoglund et al. 2012, 2014; Lazaridis et al. 2014; Günther et al. 2018). Whereas U5a was mostly distributed in Northern and Eastern Europe during the Mesolithic Stone Age, haplogroup U5b reached its highest frequencies in Central and Southern Europe (Chandler 2005; Bramanti et al. 2009; Hervella et al. 2012; Sánchez-Quinto et al. 2012; Bollongino et al. 2013; Fu et al. 2013; Lazaridis et al. 2014; Posth et al. 2016). Lineage U4 diverged somewhat later, approximately 17,000–20,900 ya (Soares et al. 2009; Behar et al. 2012), and subsequently occupied same geographical areas as U5a (Malmström et al. 2009, 2015; Skoglund et al. 2012, 2014; Der Sarkissian et al. 2013; Saag et al. 2017; Günther et al. 2018; Mittnik et al. 2018).

Starting around 10,000 ya new populations, and hence also new mitochondrial lineages, with Near Eastern origin spread to Europe leading to drastic decline in the frequencies of U sublineages (Haak et al. 2005; Bramanti et al. 2009; Brotherton et al. 2013). Along with the extensive population migration during the Bronze Age, particularly U5a and in lesser degree also U4, experienced a new expansion with the rapid westward spread of the Yamnaya culture (Keyser et al. 2009; Der Sarkissian et al. 2013; Wilde et al. 2014; Allentoft et al. 2015; Haak et al. 2015; Mathieson et al. 2015; Pilipenko et al. 2015; Mittnik et al. 2018). The frequency of U5a reached up to 23% among Yamnaya (Wilde et al. 2014; Allentoft et al. 2015; Haak et al. 2015; Mathieson et al. 2015). Nowadays, U5a and U5b are spread throughout Europe: U5a has its highest frequency in northeastern Europe (8–10% [Malyarchuk and Derenko 2001; Loogväli et al. 2004; Lappalainen et al. 2008]), whereas U5b displays maximum densities in Saami (48% [Sajantila et al. 1996; Tambets et al. 2004]), Basques (15% [Cardoso et al. 2011]), and Finns (14% [Finnilä et al. 2001]). U2 and U4 are distributed in Europe with much lower frequencies, except in the Volga-Ural region, where U4 peaks in Komi-Zyryans (24%) and U2 in Udmurts (10%) (Bermisheva et al. 2002).

The notable substitution rate differences observed among U5a and U5b have an apparent influence also on the divergence time estimates. Whereas in the previous studies (Soares et al. 2009; Malyarchuk et al. 2010; Behar et al. 2012) the divergence time estimates for U5a and U5b have been relatively alike within each study, here the divergence of U5b appears to have taken place considerably earlier than that of U5a (29,700 and 17,700 ybp, respectively). This suggests that U5b evolved already during the pre-Last Glacial Maximum similarly to U2 and U8, whereas U5a emerged later, during the LGM. Although genotyped human remains from the pre-LGM exist (Krause et al. 2010; Benazzi et al. 2015; Posth et al. 2016), the number of individuals studied is still small and more pre-LGM samples would be needed to directly confirm the age of U5b.

### Range Expansion a Plausible Explanation for Higher U5a Rate

The proposed scenario suggests that higher substitution rate for U5a and U4 is caused by population expansion. This interestingly corresponds with the observations from the Y-chromosomal lineages: accelerated mutation rates have been obtained for haplogroup R1b (Dupuy et al. 2004; Claerhout et al. 2018), which has also been associated with the vast Bronze Age population spread into Europe (Haak et al. 2015; Batini and Jobling 2017). This temporal and demographic resemblance between the lineages of different genetic markers lends even more support for the population dynamics inducing the haplogroup-specific substitution rates.

The role of population range expansions in creating the substitution rate differences observed in our data and in the Y-chromosomal studies above gain further support from theoretical studies. Since the seminal study by Edmonds et al. (2004), a number of simulation studies have shown that range expansion can have a marked effect on the gene pools (for review see Excoffier et al. 2009 and Peischl et al. 2016). In short, mutations occurring at the front of the expansion wave have a much higher probability to survive and to become fixed in the newly established populations than mutations outside this front. This phenomenon has been termed “surfing” (Klopfstein et al. 2006), and it would offer a plausible explanation for higher substitution rate especially in the subhaplogroup U5a. Indeed, Klopfstein et al. (2006) state that “times of population range expansions are very important evolutionary periods, where mutations could pre-dominantly accumulate, potentially contributing to well-known lineage-specific differences in rates of evolution.” Accelerated rate of phenotypic evolution associated with range expansions was observed already by Darwin, and shown in genomes on virtually all levels of taxonomy from phyla to intraspecific populations or lineages (see Bromham and Woolfit 2004 and references therein). A lucid example comes from a genomic assessment of *Yersinia pestis,* analyzed from samples dated to span the periods of extremely rapid epidemic spread, including the Black Death in Europe in AD 1347–1351. The fastest evolving *Y. pestis* lineages, identified to belong to strains that caused the three distinct plague epidemics, had up to 40 times higher substitution rates than the slowly-evolving, geographically more stagnant lineages (Spyrou et al. 2019).

## Conclusion

The recent decade has seen a massive increase in the amount of DNA sequence data including also ancient sequences produced from radiocarbon-dated archaeological samples. Population genetic and phylogenetic assessments from these data have given new insights into the history of a wide array of taxa, including our species. However, the reliability of many genetic inferences and timing of the evolutionary events rely on the accuracy of molecular rates assumed in the analyses.

Here we have assessed lineage-specific variation among the human mitochondrial genome lineages by focusing on the haplogroup U. Full good-quality mtDNA genomes were retrieved from repositories, including data both from contemporary and ^14^C-dated archaeological samples for which estimates of molecular rates were obtained by tip-dating method in Bayesian framework. The results show time-dependency of the mutation rate estimates as well as substantial variation in the rate of evolution among the human mtDNA subhaplogroups, especially between U4/U5a and U5b. Leaning on the theoretical and empirical evidence on the effects of range expansion on genetic variation we propose that the faster substitution rates in subhaplogroup U4 and U5a observed in this study can be at least partly appointed to the massive spread of the Yamnaya culture in the Bronze Age.

## Materials and Methods

### Data Analysis Methods

All the materials used in this study were obtained from publicly available sources. Altogether we collected 535 complete mitochondrial sequences from subhaplogroups of U. These data comprised 234 ancient genomes and 301 contemporary genomes. For details, see table 1 and supplementary tables S1 and S2, Supplementary Material online.

Haplogroup assessment for all the sequences was performed with HaploGrep2 (Weissensteiner et al. 2016) and sequences were further aligned with MAFFT v7 (Katoh and Standley 2013). In MAFFT, the alignment was done with an iterative refinement method and by assuming the default scoring matrix based upon Kimura’s two-parameter model. The best-fit substitution model for each data set was determined with bModelTest (Bouckaert and Drummond 2017).

As the only calibration method used in this study was tip-date calibration, we first evaluated with TempEst (Rambaut et al. 2016) if the sampling dates were sufficient enough to produce a temporal signal. In TempEst for the root-to-tip reconstruction, R squared was selected as a best-fit root position method. For all the subsequent phylogenetic analyses we used BEAST v2.6.2 software package (Bayesian evolutionary analysis by sampling trees) (Bouckaert et al. 2019). For every analysis, the whole mtDNA sequences were treated as one partition and the substitution models for different data sets were set according to supplementary table S3, Supplementary Material online. To model the rate heterogeneity, discrete gamma distribution (+Γ) was used with a number of shape parameter categories of 4. Since, in all the data sets, the majority of the sequence positions did not contain polymorphisms, the proportion of invariants was additionally taken into account (pInv). As a molecular clock model, the uncorrelated lognormal relaxed clock was used to allow variation in the substitution rates between the lineages (Drummond et al. 2006). In fact, assumption of a constant molecular clock throughout time of interest has shown to be questionable especially when recent evolutionary processes, such as the human past, are investigated (Ho and Larson 2006). As suggested in Drummond and Bouckaert (2015) we used the lognormal relaxed clock with the following prior on ucldStdev: gamma distribution with shape = 0.5396 and scale = 0.3819. This prior places 50% of the probability mass below value 0.1.

The non-parametric coalescent-based Bayesian skyline was used as a tree prior, since it does not require any prior assumptions about the population’s demographic past. Moreover, it has been demonstrated that the assumed demographic model has no significant impact on the substitution rates (Ho et al. 2007).

All the ancient samples included in this study were ^14^C-dated and this information was used as tip-calibration to estimate the molecular rates and hence also the evolutionary timescale. Sample-dating error has been shown to introduce only a limited impact on the substitution and divergence time estimates (Molak et al. 2013, 2015; Rieux et al. 2014) and hence only the mean values of the ^14^C distributions were used. For consistency, all the radiocarbon dates were calibrated with Oxcal 4.3 (Bronk Ramsey 2009) IntCal 13 as a calibration curve (Reimer et al. 2013). No additional prior assumptions were made.

Length for the Markov chain was set to 15 or 100 million steps, depending on the number of sequences in the data set analyzed. For each analysis, three independent runs were performed and further inspected in Tracer v1.7.1 (Rambaut et al. 2018). The consistency of the three independent runs was evaluated by comparing the posterior distributions for each parameter by eye. Parallel runs were then further combined with LogCombiner, part of the BEAST package, and subsequently the effective sample sizes for each parameter were confirmed to be above 200, after removing the first 10% of the chain as burn-in. The maximum clade credibility tree was chosen with TreeAnnotator, also provided in the BEAST software package. As in Tracer, the first 10% of the steps were discarded as burn-in. Node heights presented in the figures are median heights. For the tree visualization, FigTree v1.4.1 (http://tree.bio.ed.ac.uk/software/figtree/) was used.

To replicate our results with an independent method we used Least-Squares Dating (LSD2) method v.1.9.9 (To et al. 2016) implemented in IQ-Tree 2.0.3 (Minh et al. 2020). Least-Squares Dating was performed for scenarios A and B. Following command was used: *input.fasta -m HKY + I+G -B 1000 –date TAXNAME –date-ci 100 –date-options -l 0,* except for U5b for which the substitution model GTR + I + G was used.

As the diversity within the subhaplogroups could also be differentially affected by selection, we assessed signals of selection in 12 coding gene regions spanning 10,810 bp (65%) of the mtDNA genome. Due to its L-strand translation, 525 bp of the *ND6* gene were not included in the analysis. For inferring the coding regions and reading frames correctly, all sequences were forced to an rCRS alignment (MAFFT option – keeplength). The selection analyses were performed using the Z-test implemented in MEGA-X software v.10.1.7 (Kumar et al. 2018). The average number of nonsynonymous (*d_N_*) and synonymous (*d_S_*) substitutions (per S/N sites) were estimated using the Pamilo–Bianchi–Li method (Pamilo and Bianchi 1993; Li 1993) (Kimura two-parameter model), assuming pairwise deletion of ambiguous sites. The variances of *d_S_* and *d_N_* were estimated using 500 bootstrap replicates. For all data sets (A–C) within the subhaplogoups U2, U4, U5a, and U5b the probability of rejecting the null hypothesis of strict neutrality (*d_N_* = *d_S_*) were estimated.

## Supplementary Material

Supplementary data are available at *Genome Biology and Evolution* online.

## Supplementary Material

evac097_Supplementary_Data

## Data Availability

The data underlying this article is available from GenBank at https://www.ncbi.nlm.nih.gov/genbank/and from Ancient mtDNA database (AmtDB) at https://amtdb.org/. For GenBank accession numbers and AmtDB identifiers required to access the sequences see supplementary tables S1 and S2, Supplementary Material online.
