# Supplementary material for: Variation in the Substitution Rates among the Human Mitochondrial Haplogroup U Sublineages
Source: Genome Biol Evol. 2022 Jun 22;14(7):evac097. doi: 10.1093/gbe/evac097 (PMC9250076; doi:10.1093/gbe/evac097)
Supplement: evac097_Supplementary_Data [file evac097_supplementary_data.zip › SupplementaryInformation_Oversti_Palo_GBE-210710.R2_Revision.pdf]

Variation in the substitution rates among the human mitochondrial haplogroup U sublineages

Sanni Översti, Jukka U. Palo

Author for Correspondence: Sanni Översti, sanni.oversti@helsinki.fi

Supplementary Information

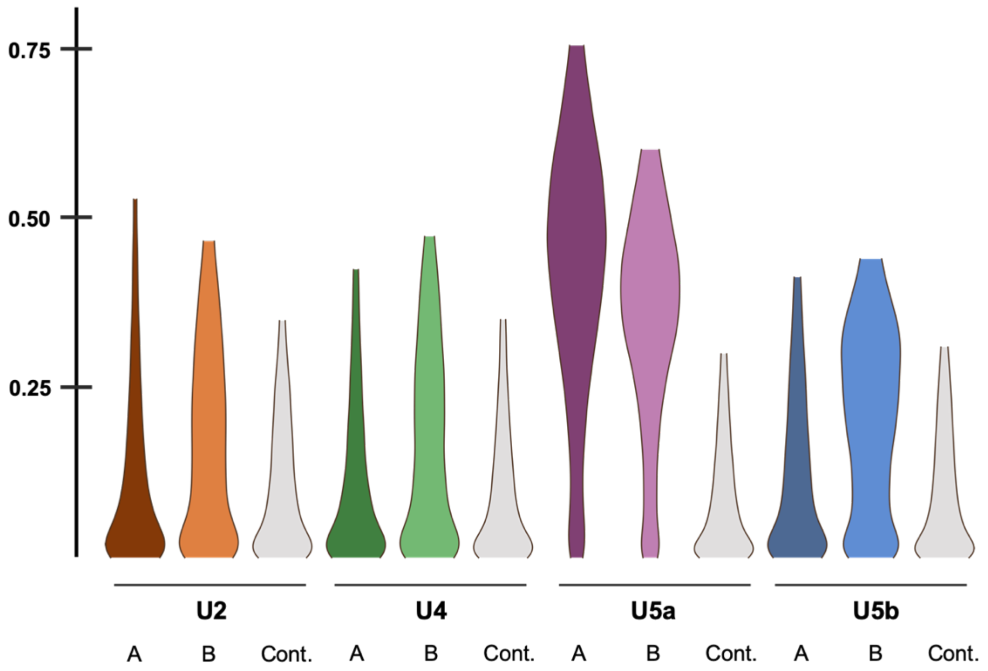

Supplementary Figure S1. Marginal posterior distributions for coefficient of variation estimates with 95% highest posterior density intervals. For each subhaplogroup coefficient of variation values are presented for scenarios A and B (aDNA and aDNA+contemporary) as well as for contemporary-only data ('Cont.'). Whereas for U2, U4 and U5b the marginal posterior distributions for scenarios A and contemporary-only are nearly identical within haplogroups, for U5a substantial differences arise.
